# Glycyrrhizin (GLY) and glycyrrhetinic acid (GA) as potential multi-target antimicrobials: mechanisms, biofilm disruption, and synergy against drug-resistant pathogens

**DOI:** 10.3389/fmicb.2026.1861692

**Published:** 2026-06-22

**Authors:** Kumar D. Gahlot

**Affiliations:** 1 Department of Molecular Biology, Umeå University, Umeå, Sweden; 2 Umeå Centre for Microbial Research (UCMR), Umeå University, Umeå, Sweden

**Keywords:** biofilms, glycyrrhetinic acid (GA), glycyrrhizin (GLY), host–pathogen interactions, multi-target antimicrobials, natural products, triterpenoids

## Abstract

Antimicrobial resistance (AMR) is a major global health challenge of the 21*^st^* century, driven by multidrug-resistant pathogens, persistent biofilm-associated infections, and a dwindling pipeline of new antibiotics. These limitations have renewed interest in natural products with multi-target antimicrobial activity. *Glycyrrhiza glabra*, a medicinal herb, produces glycyrrhizin (GLY), a triterpenoid saponin, and its active metabolite, glycyrrhetinic acid (GA), both of which exhibit antibacterial, antiviral, and antibiofilm activity. These compounds act through coordinated mechanisms, including disrupting microbial membranes, inhibiting metabolic pathways, modulating efflux systems, suppressing biofilm structure, and regulating host inflammatory responses. Their ability to enhance antibiotic efficacy further supports their role as adjunctive therapeutic agents. This review critically synthesizes current knowledge on the chemical properties, molecular mechanisms of action, pathogen-specific activity, biofilm interference, pharmacokinetics, safety considerations, and translational potential of these triterpenoids. Collectively, the available data could position *G. glabra*-derived triterpenoids as promising adjunctive candidates for next-generation multi-target antimicrobials.

## Introduction

Antimicrobial resistance (AMR) is a major global health challenge, driven by the rapid emergence and dissemination of multidrug-resistant pathogens (GBD 2021 Antimicrobial Resistance Collaborators, 2024). The spread of resistance is facilitated by horizontal gene transfer, plasmid-mediated mechanisms, and adaptive bacterial responses that enable survival under antimicrobial pressure (Negeri et al., 2023; Patkowski et al., 2023). Conjugative systems such as the F-pilus play a critical role in this process, enhancing both resistance gene transfer and biofilm formation, thereby accelerating microbial persistence and evolution (Patkowski et al., 2023). In parallel, regulatory networks such as the Cpx envelope stress response coordinate resistance, virulence, and environmental adaptation, including lipid A remodeling and tolerance to last-resort antimicrobials (Gahlot et al., 2024; Thanikkal et al., 2019).

Biofilm formation further complicates antimicrobial therapy. These structured microbial communities (biofilms), embedded within extracellular polymeric substances, provide both physical protection and metabolic advantages, enabling survival under stress conditions (Ciofu et al., 2022). Our recent work demonstrates that Cpx-signaling also facilitates Hms-dependent biofilm formation, linking stress adaptation directly to persistence (Gahlot et al., 2022b). Structural and regulatory determinants such as pili/fimbriae assembled *via* the chaperone–usher pathway, virulence regulators (e.g., Caf1R), and secretion systems additionally contribute to adhesion, colonization, and host–pathogen interactions (Gahlot et al., 2021, 2022a; Mangu et al., 2026). Together, these processes highlight the complexity of microbial pathogenesis and the limitations of single-target antimicrobial strategies.

Beyond pathogenicity, microbial adaptability also extends to environmental and ecological contexts. Engineered bacterial systems have demonstrated plasticity in stress responses and metabolic functions, such as copper accumulation in *Escherichia coli*, underscoring the interconnected nature of microbial physiology, adaptation, and survival (Gahlot et al., 2020). These insights, combined with studies on anti-infective strategies targeting *Yersinia* virulence systems, emphasize the need for therapeutics that can simultaneously target multiple aspects of microbial pathophysiology (Gahlot et al., 2026).

**FIGURE 1:**
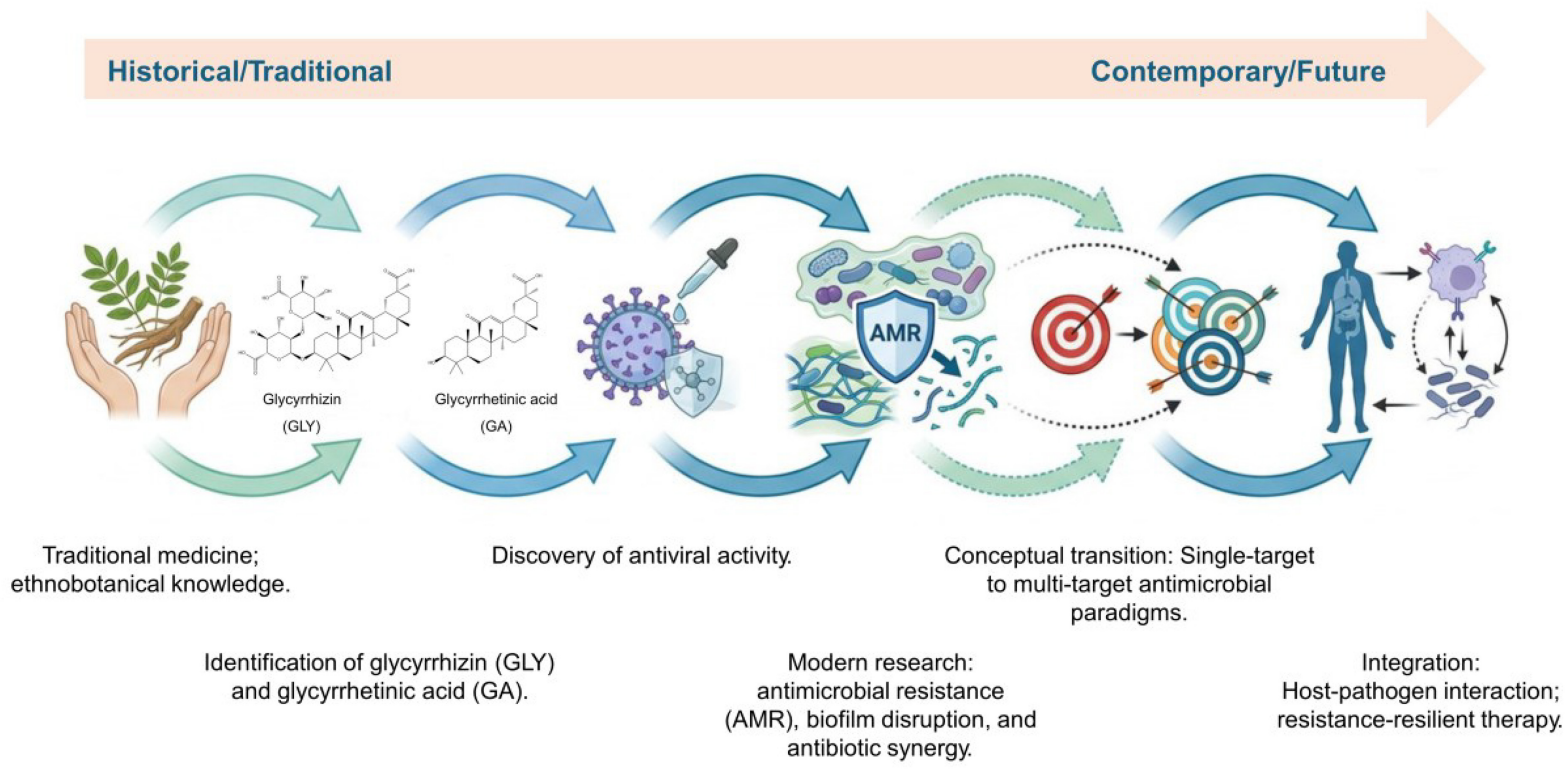
Historical and conceptual evolution of *Glycyrrhiza glabra* research. Schematic timeline illustrating the progression of *G. glabra* from traditional medicinal use to modern antimicrobial research. The Figure integrates ethnobotanical knowledge with contemporary microbiological and pharmacological discoveries, highlighting key milestones including the identification of glycyrrhizin (GLY) and glycyrrhetinic acid (GA), early antiviral applications, and recent advances in antimicrobial resistance, biofilm disruption, and antibiotic synergy. The Figure also conceptualizes the transition from single-target to multi-target antimicrobial paradigms, positioning GLY and GA within modern frameworks of host–pathogen interaction and resistance-resilient therapy for infectious disease research.

In response to these challenges, natural products have re-emerged as promising sources of multi-target antimicrobial agents. Among these, *Glycyrrhiza glabra*–derived triterpenoids have gained significant attention (Figure 1) due to their broad pharmacological properties (Rafiq et al., 2026). Glycyrrhizin (GLY), the principal active compound, is metabolized by intestinal microbiota into glycyrrhetinic acid (GA), a more lipophilic and biologically active metabolite (Krahenbuhl et al., 1994; Ploeger et al., 2001). Unlike conventional antibiotics that typically act on single molecular targets, these triterpenoids exhibit integrated antimicrobial activity involving membrane disruption, metabolic modulation, biofilm interference, efflux, quorum-sensing inhibition, and host immune regulation (Chen L. et al., 2024; Chen R. et al., 2024; Long et al., 2013; Rafiq et al., 2026; Semwal et al., 2025; Weaver et al., 2022).

Experimental studies demonstrate that GA reduces bacterial viability and virulence, while GLY enhances antibiotic efficacy and modulates host inflammatory pathways, including NF-κB signaling and glucocorticoid regulation (Hazlett et al., 2019; Long et al., 2013; Yu et al., 2026). These multi-layered effects are consistent with broader advances in antimicrobial strategies, such as nanomaterial-based approaches. For instance, chlorhexidine-loaded zinc nanoparticles have shown enhanced antibacterial efficacy against *Streptococcus pneumoniae*, highlighting the benefit of combining structural and biochemical targeting mechanisms (Kumar et al., 2025).

In addition to direct antimicrobial effects, these triterpenoids influence microbial metabolism, gene expression, and community behavior, as evidenced by changes in bacterial metabolomic profiles, transcriptional regulation, and microbiota composition, suggesting broader roles in microbiome modulation and ecological balance (Tian et al., 2025; Weaver et al., 2022; Wu et al., 2025; Yu et al., 2024). Such properties align with emerging therapeutic paradigms that integrate pathogen targeting with host-directed and microbiome-informed strategies.

Collectively, these findings highlight the need for comprehensive antimicrobial approaches that address the complexity of microbial resistance, biofilm formation, and host interactions. Both GLY and GA exemplify multifunctional agents capable of targeting structural, metabolic, and regulatory pathways while modulating host responses. This systems-level activity positions them as promising candidates for next-generation antimicrobial development to combat antimicrobial resistance and persistent biofilm-associated infections. Accordingly, a more nuanced evaluation of both the opportunities and limitations of these compounds is essential to guide their development as clinically viable antimicrobial adjuvants within precision medicine frameworks.

## Chemical and mechanistic basis of antimicrobial activity

**FIGURE 2:**
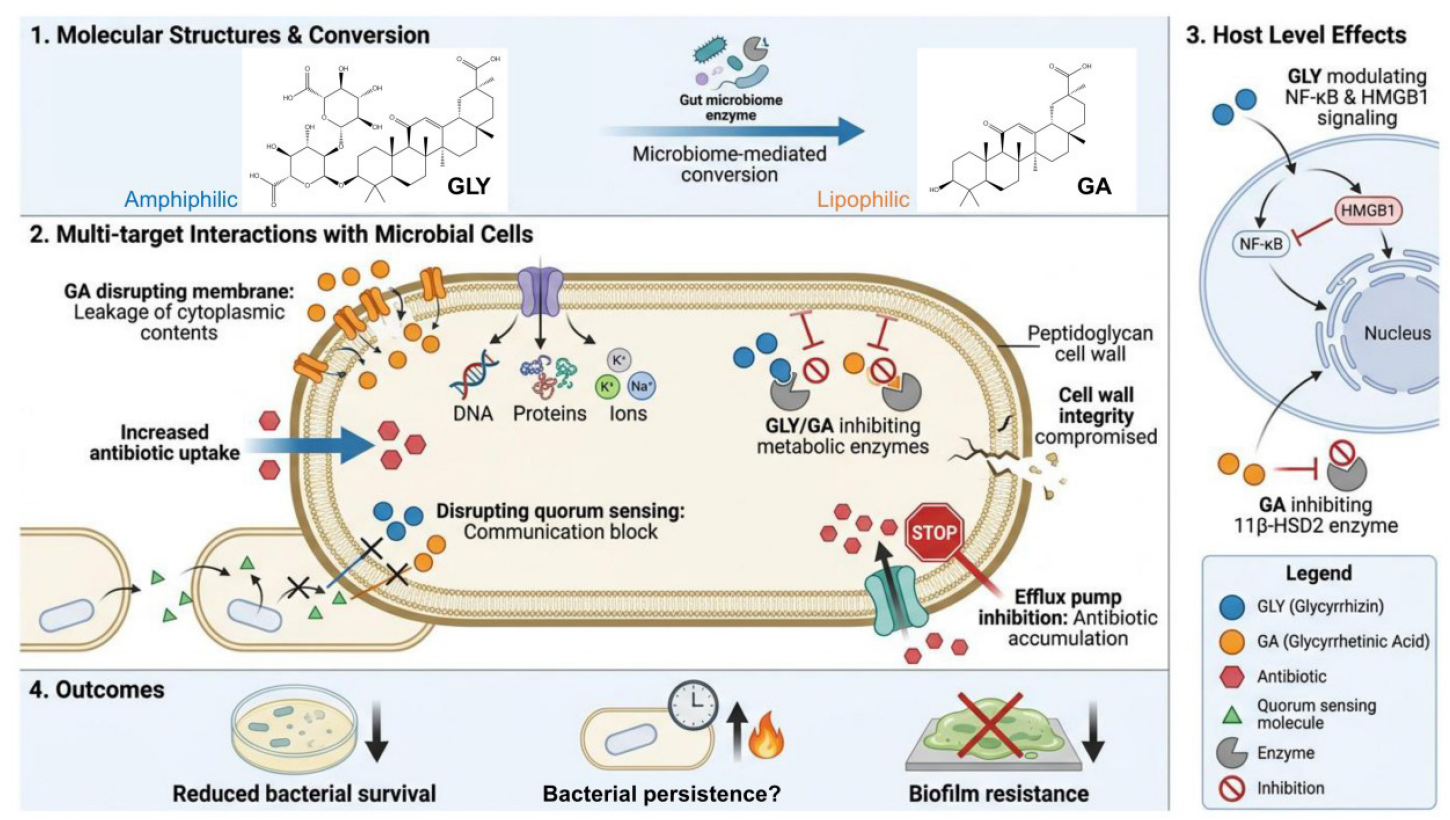
Chemical and mechanistic basis of glycyrrhizin (GLY) and glycyrrhetinic acid (GA)–mediated antimicrobial activity. This Figure illustrates the amphiphilic structure of GLY and its more lipophilic derivative, GA (microbiome-mediated conversion). These properties enable multi-target interactions with microbial cells. GA disrupts membrane integrity, leading to leakage of intracellular components and increased antibiotic uptake. Both triterpenoids inhibit metabolic enzymes and affect cell wall integrity, and they also disrupt quorum sensing and inhibit efflux pumps, thereby reducing virulence and resistance. At the host level, GLY modulates NF-κB and HMGB1 signaling, and GA inhibits 11β-HSD2. Together, these mechanisms reduce bacterial survival, persistence, and biofilm-associated resistance.

Glycyrrhizin is an amphiphilic triterpenoid composed of a hydrophobic aglycone conjugated to hydrophilic glucuronic acid residues, enabling interaction with both aqueous environments and lipid membranes (Figure 2 and Table 1). Following oral administration, GLY is hydrolysed by intestinal microbiota to GA, a more lipophilic and membrane-permeable metabolite with enhanced biological activity (Ploeger et al., 2001; Rasool and Dar, 2025).

**TABLE 1:** Comparative analysis of glycyrrhizin (GLY) vs. glycyrrhetinic acid (GA).

Parameter	Glycyrrhizin (GLY)	Glycyrrhetinic acid (GA)	References
**Chemical nature**	Hydrophilic triterpenoid saponin (glycoside)	Lipophilic aglycone metabolite	Ploeger et al., 2001
**Primary role**	Prodrug; indirect antimicrobial/immunomodulator	Active antimicrobial effector molecule	Ploeger et al., 2001
**Pharmacokinetics (absorption and metabolism)**	Poorly absorbed; converted by gut microbiota (β-glucuronidase)	Readily absorbed after conversion; higher permeability	Ploeger et al., 2001
**Bioavailability determinants**	Highly dependent on microbiome composition	More stable bioavailability post-conversion	Ploeger et al., 2001
**Distribution and retention**	Limited systemic distribution	Broad tissue distribution; enterohepatic circulation prolongs exposure	Ploeger et al., 2001
**Core antimicrobial mechanisms**	Weak direct effects; immune modulation	Strong membrane disruption, enzyme inhibition, metabolic suppression	Long et al., 2013
**Host-directed effects**	Strong immunomodulation (anti-inflammatory signalling)	Moderate host modulation	Ma et al., 2026
**Enzyme targeting**	Indirect modulation of host pathways	Direct inhibition of bacterial enzymes and 11β-HSD2	Van Uum et al., 2002
**Biofilm inhibition**	Moderate; mainly indirect via signaling	Strong; reduces EPS, disrupts structure, inhibits metabolism	Dewake et al., 2021
**MIC/antibacterial potency**	Higher MIC (weaker)	Lower MIC; stronger antibacterial activity	Long et al., 2013
**Antibiotic synergy**	Enhances immune-mediated antibiotic action	Enhances drug uptake and retention	Hazlett et al., 2019
**Mechanism of synergy**	Reduces inflammation; improves host-driven clearance	Increases permeability; inhibits efflux; disrupts resistance pathways	Alav et al., 2018; Ren et al., 2024
**Resistance modulation**	Indirect via ecological/immune mechanisms	Direct via membrane and metabolic disruption	Hazlett et al., 2019
**Safety profile**	Generally safe; depends on conversion	Major contributor to toxicity at high dose	Van Uum et al., 2002; Cao et al., 2022
**Key toxicity mechanism**	Requires conversion to active metabolite	Inhibits 11β-HSD2 → pseudoaldosteronism (hypertension, hypokalemia)	Van Uum et al., 2002; Cao et al., 2022
**Clinical evidence**	Adjunct therapeutic effects demonstrated	Associated with resistant hypertension and endocrine imbalance	Awad et al., 2020; Hung et al., 2017; Ma et al., 2026; Orlent et al., 2006; Takahashi et al., 2019
**Limitations**	Microbiome-dependent variability; weaker standalone efficacy	Dose-dependent toxicity; endocrine side effects	Awad et al., 2020; Hung et al., 2017; Ma et al., 2026; Orlent et al., 2006; Takahashi et al., 2019
**Translational potential**	Strong in host-directed therapy and precision medicine	Strong antimicrobial potential but requires controlled delivery	Ma et al., 2026
**Overall functional role**	Immunomodulatory and synergistic facilitator	Principal antimicrobial and anti-biofilm effector	–

As illustrated in Figure 2, the antimicrobial activity of GLY and GA is driven by their structural properties, which support multi-target interactions with microbial cells. A primary mechanism is membrane disruption: GA integrates into lipid bilayers, altering membrane integrity and permeability. This leads to leakage of intracellular components, dissipation of ion gradients, and ultimately bacterial cell death (Long et al., 2013). Increased membrane permeability also facilitates antibiotic uptake.

In addition to membrane effects, these triterpenoids interfere with intracellular enzyme systems. Disruption of membrane homeostasis impairs enzymatic function and metabolic flux, while experimental evidence indicates direct and indirect inhibition of enzymes involved in central metabolism and biosynthetic pathways (Dewake et al., 2021; Sarkar et al., 2023). GA has also been shown to affect cell wall dynamics, including peptidoglycan integrity and associated enzymatic processes, further compromising bacterial structural stability (Long et al., 2013). Both triterpenoids additionally modulate bacterial regulatory systems. They disrupt quorum-sensing pathways, reducing virulence factor expression and coordinated biofilm maturation (Alav et al., 2018). Interference with efflux pump activity is another key mechanism, as inhibiting drug efflux increases intracellular antibiotic accumulation and enhances antimicrobial efficacy (Alav et al., 2018; Ren et al., 2024).

Beyond direct microbial effects, GLY interacts with host signaling pathways. It modulates inflammatory responses through pathways such as NF-κB and HMGB1, while GA inhibits 11β-hydroxysteroid dehydrogenase type 2, influencing glucocorticoid-mediated immune regulation (Mollica et al., 2007; Semwal et al., 2025; Suzuki et al., 1998; Van Uum et al., 2002).

Collectively, both triterpenoids exert antimicrobial activity through an integrated set of mechanisms, including membrane disruption, enzyme inhibition, modulation of cell wall dynamics, interference with efflux pumps, and modulation of host immune signaling (Figure 2). This multifaceted mode of action reduces bacterial survival, virulence, and persistence, particularly under biofilm and antibiotic stress conditions.

## Pathogen-specific antimicrobial activity

**FIGURE 3:**
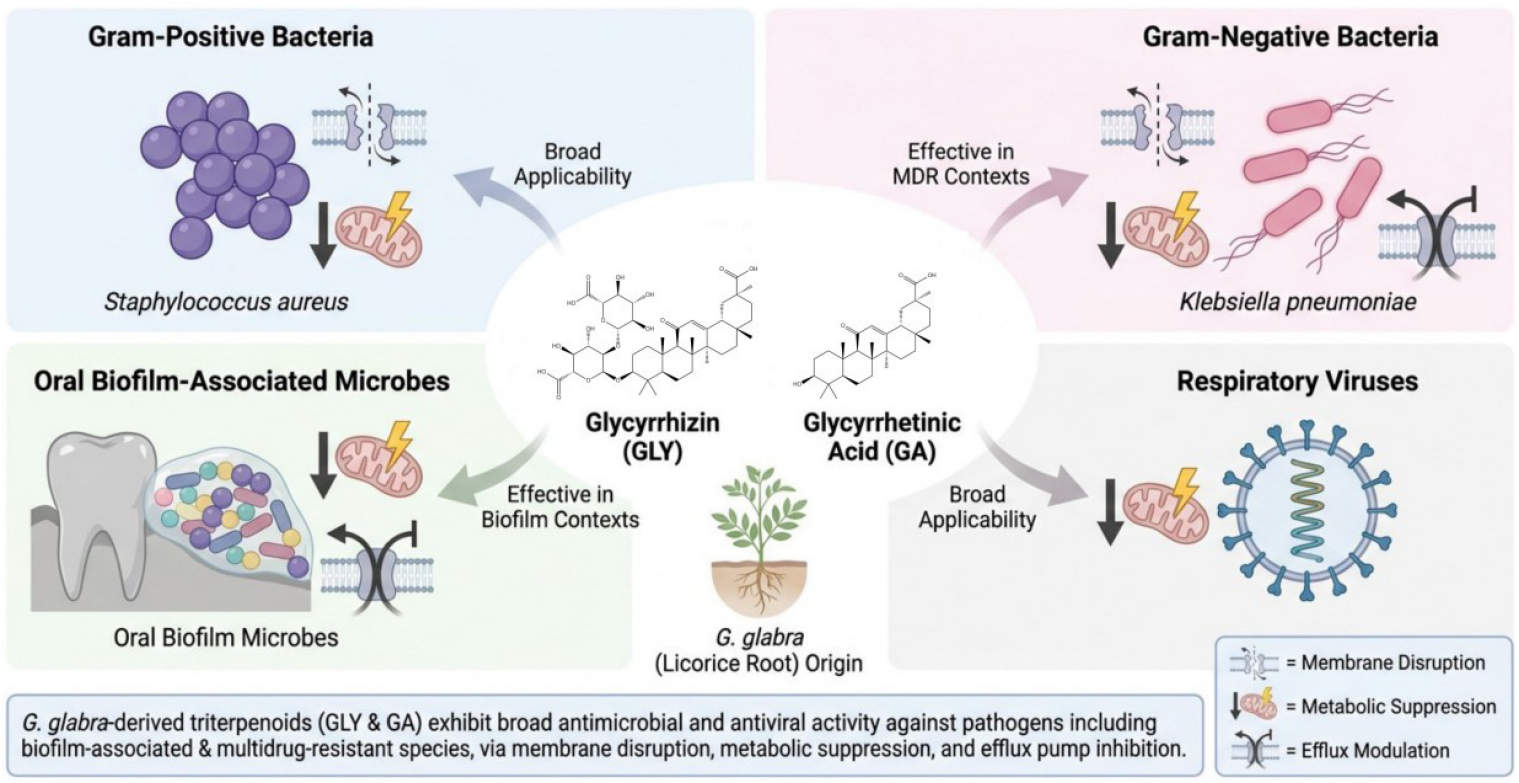
Pathogen spectrum targeted by glycyrrhizin (GLY) and glycyrrhetinic acid (GA). This Figure summarizes the antimicrobial and antiviral spectrum of GLY and GA across key pathogen groups. For example, Gram-positive bacteria (e.g., *Staphylococcus aureus*), Gram-negative pathogens (e.g., *Klebsiella pneumoniae*), oral biofilm-associated microbes, and respiratory viruses. Mechanistic overlays indicate dominant modes of action, including membrane disruption, metabolic suppression, and efflux modulation. The Figure emphasizes the broad applicability of these triterpenoids in diverse infectious contexts, including biofilm-associated and multidrug-resistant infections.

Both triterpenoids exhibit broad-spectrum antimicrobial activity against diverse microbial groups, including Gram-positive and Gram-negative bacteria, biofilm-associated pathogens, and viruses (Figure 3). Their activity reflects a combination of direct antimicrobial effects and indirect physiological disruption, which together enhance therapeutic efficacy. Among Gram-positive bacteria, *Staphylococcus aureus* is one of the most extensively studied. Experimental studies demonstrate that GA significantly inhibits bacterial growth, reduces virulence factor expression, and disrupts metabolic activity in methicillin-resistant strains (Long et al., 2013; Rijo et al., 2024; Weaver et al., 2022). These effects are particularly relevant in chronic infections, including wound and implant-associated diseases, where *S. aureus* plays a central role.

Gram-negative pathogens, including *Klebsiella pneumoniae*, also exhibit susceptibility to GA. In these organisms, GA compromises outer membrane integrity and interferes with efflux-mediated resistance mechanisms, thereby enhancing antimicrobial susceptibility (Chen L. et al., 2024; Chen R. et al., 2024; Ham et al., 2025; Irani et al., 2010; Leus and Zgurskaya, 2025; Rafiq et al., 2026). This dual impact on structural barriers and resistance pathways underscores GA’s versatility against structurally diverse bacterial groups.

Beyond bacteria, GLY exhibits inhibitory activity against oral microbial communities associated with dental plaque and periodontal disease (AlDehlawi and Jazzar, 2023; Ham and Kim, 2023; Sun et al., 2020). This supports its application in oral health formulations. In addition, GLY demonstrates antiviral activity against respiratory viruses, including coronaviruses and influenza viruses, through inhibition of viral replication and modulation of host immune responses (Cinatl et al., 2003; Huan et al., 2021; Li et al., 2021; Tolah et al., 2021; van de Sand et al., 2021).

Importantly, pathogen susceptibility varies depending on microbial physiology and environmental conditions. Biofilm-forming organisms exhibit increased tolerance compared with planktonic cells, necessitating higher concentrations or combination therapies (Ciofu et al., 2022; Trubenová et al., 2022). Nevertheless, both GLY and GA retain activity under these conditions, particularly when combined with conventional antibiotics (Rafiq et al., 2026; Rijo et al., 2024; Singh et al., 2023). At the mechanistic level, GA acts primarily through membrane disruption and metabolic inhibition, while GLY contributes through immunomodulation and metabolic reprogramming (Buder et al., 2022; Jin et al., 2022; Liu et al., 2019; Semwal et al., 2025; Weaver et al., 2022). This dual functionality enables these compounds to target multiple microbial systems simultaneously.

The broad-spectrum activity of these triterpenoids highlights their potential as versatile antimicrobial agents. Their effectiveness across different microbial taxa, combined with their ability to modulate resistance pathways, positions them as promising candidates for addressing complex infections.

## Integrated biofilm disruption, antibiotic synergy, and host immune modulation

**FIGURE 4:**
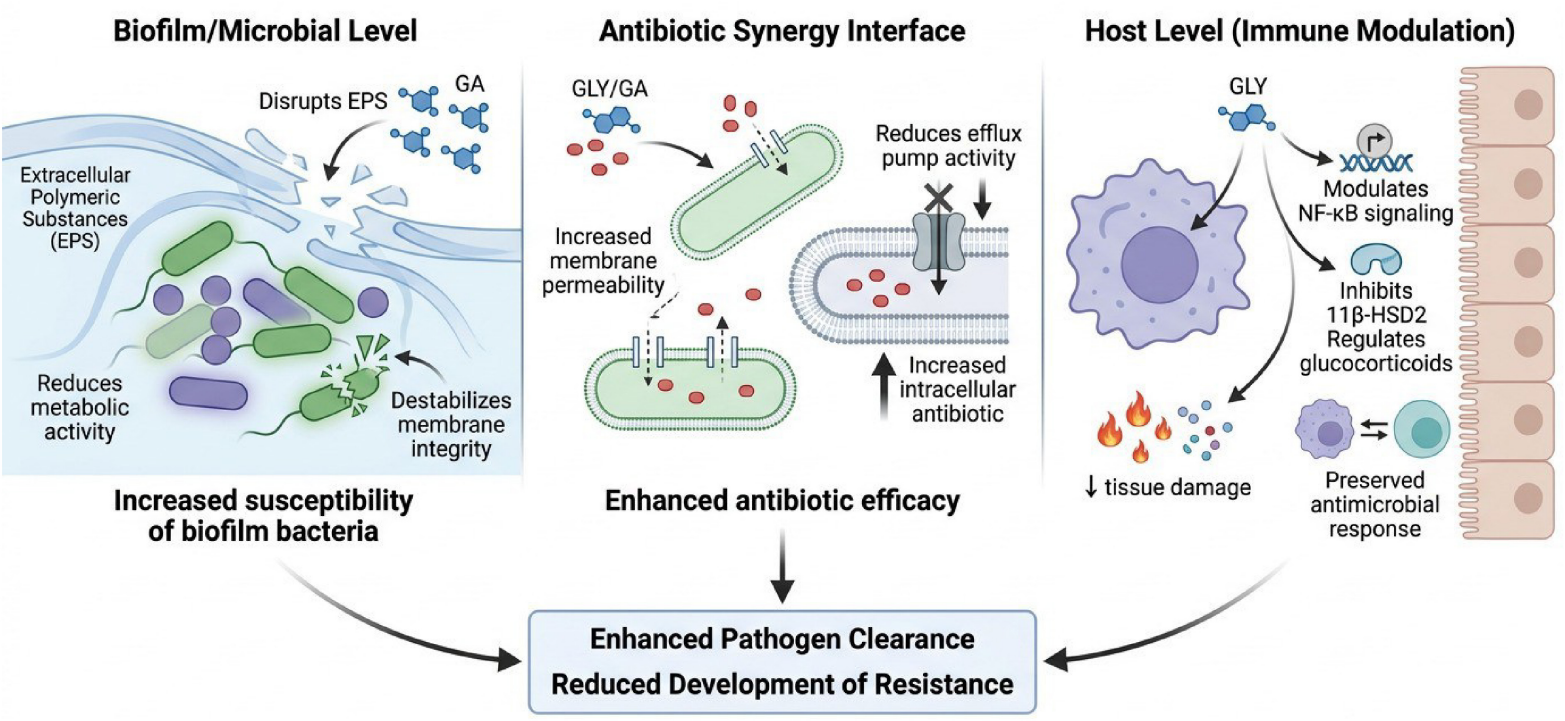
Integrated model of biofilm disruption, antibiotic synergy, and host immune modulation. This conceptual framework illustrates the multi-layered antimicrobial action of glycyrrhizin (GLY) and glycyrrhetinic acid (GA) across microbial and host systems. At the biofilm level, GA disrupts extracellular polymeric substances, reduces metabolic activity, and destabilizes membrane integrity, thereby increasing the susceptibility of biofilm-embedded bacteria. Simultaneously, GLY and GA enhance antibiotic efficacy by increasing membrane permeability, reducing efflux pump activity, and improving intracellular drug accumulation. At the host level, GLY modulates key inflammatory pathways, including NF-κB signaling and glucocorticoid regulation via 11β-HSD2 inhibition, reducing tissue damage while preserving antimicrobial immune responses. Arrows illustrate the integration of these processes, demonstrating how structural disruption, metabolic suppression, and immune modulation converge to enhance pathogen clearance and reduce the development of resistance.

Biofilms are key drivers of antimicrobial resistance, providing structural protection and facilitating metabolic adaptation that enhances bacterial persistence (Alav et al., 2018; Ciofu et al., 2022; Gahlot et al., 2022b; Patkowski et al., 2023; Ren et al., 2024; Trubenová et al., 2022). As illustrated in Figure 4, GLY and GA counteract biofilm-associated infections through an integrated network of structural, metabolic, regulatory, and host-directed mechanisms (Dewake et al., 2021; Ham and Kim, 2023; Ham et al., 2025; Rafiq et al., 2026; Weaver et al., 2022). At the biofilm level, GA destabilizes the extracellular polymeric substance matrix, resulting in reduced biomass, compromised architecture, and increased permeability (Dewake et al., 2021; Ham and Kim, 2023; Ham et al., 2025). This structural disruption is closely coupled with membrane destabilization, which enhances the susceptibility of biofilm-embedded bacteria and promotes deeper penetration of antimicrobial agents into otherwise protected regions of the biofilm.

Simultaneously, GA exerts significant metabolic effects by suppressing ATP production and enzyme activity, thereby limiting bacterial viability within biofilms (Long et al., 2013). GLY complements these actions through modulation of gene expression associated with carbon utilization and energy metabolism, reflecting a broader reprogramming of bacterial physiology (Sarkar et al., 2023). Collectively, these metabolic perturbations reduce the inherent tolerance of biofilm-associated bacteria and shift them toward a more antibiotic-sensitive state. This metabolic and structural destabilization is particularly important in chronic infections, where biofilm resilience is driven by adaptive metabolic downregulation (Ciofu et al., 2022; Trubenová et al., 2022).

At the regulatory level, GLY and GA disrupt quorum-sensing pathways that govern microbial communication, coordinated biofilm maturation, and virulence expression (Long et al., 2013; Rafiq et al., 2026). In parallel, inhibition of efflux pump activity reduces the active extrusion of antimicrobial agents, leading to increased intracellular antibiotic accumulation (Alav et al., 2018; Ren et al., 2024). These combined effects—enhanced membrane permeability, reduced efflux capacity, and impaired metabolic adaptability—form the mechanistic basis of strong antibiotic synergy. Consistent with this, experimental studies report reductions in minimum inhibitory concentrations (MICs) and restoration of antibiotic efficacy in resistant bacterial strains (Hazlett et al., 2019; Long et al., 2013).

Quantitative evidence further supports this multi-layered antimicrobial activity. GA demonstrates direct antibacterial effects against clinically relevant pathogens, with MIC values typically in the low-to-moderate micromolar range, reflecting both membrane disruption and metabolic interference (Dewake et al., 2021; Long et al., 2013). Notably, its efficacy is amplified in biofilm contexts, where suppression of extracellular polymeric substance matrix production and metabolic activity enhances the susceptibility of otherwise tolerant bacterial populations (Dewake et al., 2021; Ham et al., 2025).

Beyond direct antimicrobial effects, GLY and GA also contribute at the host level by modulating immune and inflammatory responses (Wang et al., 2022). GLY regulates key signaling pathways such as NF-κB, reducing excessive inflammation while preserving antimicrobial defense mechanisms (Liu et al., 2019). Concurrently, GA-mediated inhibition of 11β-hydroxysteroid dehydrogenase type 2 alters glucocorticoid signaling, influencing immune regulation and inflammatory balance (Van Uum et al., 2002). GLY additionally enhances innate immune responses, including oxidative stress pathways that support pathogen clearance (Sarkar et al., 2023).

Together, these interconnected processes demonstrate that these triterpenoids function as multifaceted antimicrobial adjuvants (Figure 4). By integrating biofilm disruption, metabolic suppression, efflux inhibition, antibiotic potentiation, and host immune modulation, they enhance pathogen clearance, improve antibiotic efficacy, and reduce the development of resistance, particularly in biofilm-associated and multidrug-resistant infections.

## Meta-analysis of GLY and GA: mechanisms, efficacy, and limitations

A synthesis of experimental and clinical evidence shows that both GLY and GA exhibit complementary antimicrobial activities, with GA acting as the principal active effector (Table 1). Owing to its higher lipophilicity and membrane permeability, GA displays stronger direct antibacterial effects, including membrane disruption, metabolic inhibition, and interference with biofilm integrity through reduction of extracellular polymeric substances, resulting in lower MICs and reduced bacterial viability (Dewake et al., 2021; Long et al., 2013).

Glycyrrhizin, in contrast, shows weaker direct antimicrobial activity but contributes indirectly by immunomodulation and by enhancing antibiotic efficacy. Both compounds demonstrate consistent synergy with conventional antibiotics, reflected in reduced MIC values and restored susceptibility in resistant strains. This synergy is driven by increased membrane permeability, inhibition of efflux pumps, and disruption of quorum-sensing pathways that regulate biofilm formation and virulence (Alav et al., 2018; Hazlett et al., 2019).

In biofilm contexts, GA exhibits greater efficacy due to enhanced penetration and structural disruption, whereas GLY contributes to regulatory and host-mediated effects. However, antimicrobial outcomes remain context-dependent, varying with microbial species, biofilm maturity, and environmental conditions, thereby limiting reproducibility and clinical standardization (Ciofu et al., 2022; Ploeger et al., 2001; Trubenová et al., 2022). Collectively, GA functions as the primary antimicrobial and anti-biofilm agent, whereas GLY acts as a precursor and synergistic modulator. Importantly, these context-dependent responses highlight a key limitation in the current evidence base, as antimicrobial efficacy is strongly influenced by factors such as microbial species composition, biofilm maturity, and environmental conditions (Ciofu et al., 2022; Trubenová et al., 2022). Such variability complicates direct comparison across studies and limits the standardization of dosing and treatment protocols in clinical settings (Ploeger et al., 2001). Furthermore, microbiome-dependent activation of glycyrrhizin introduces an additional layer of interindividual variability, which may significantly affect therapeutic outcomes (Chen R. et al., 2024).

## Pharmacokinetics, safety, and translational potential

**FIGURE 5:**
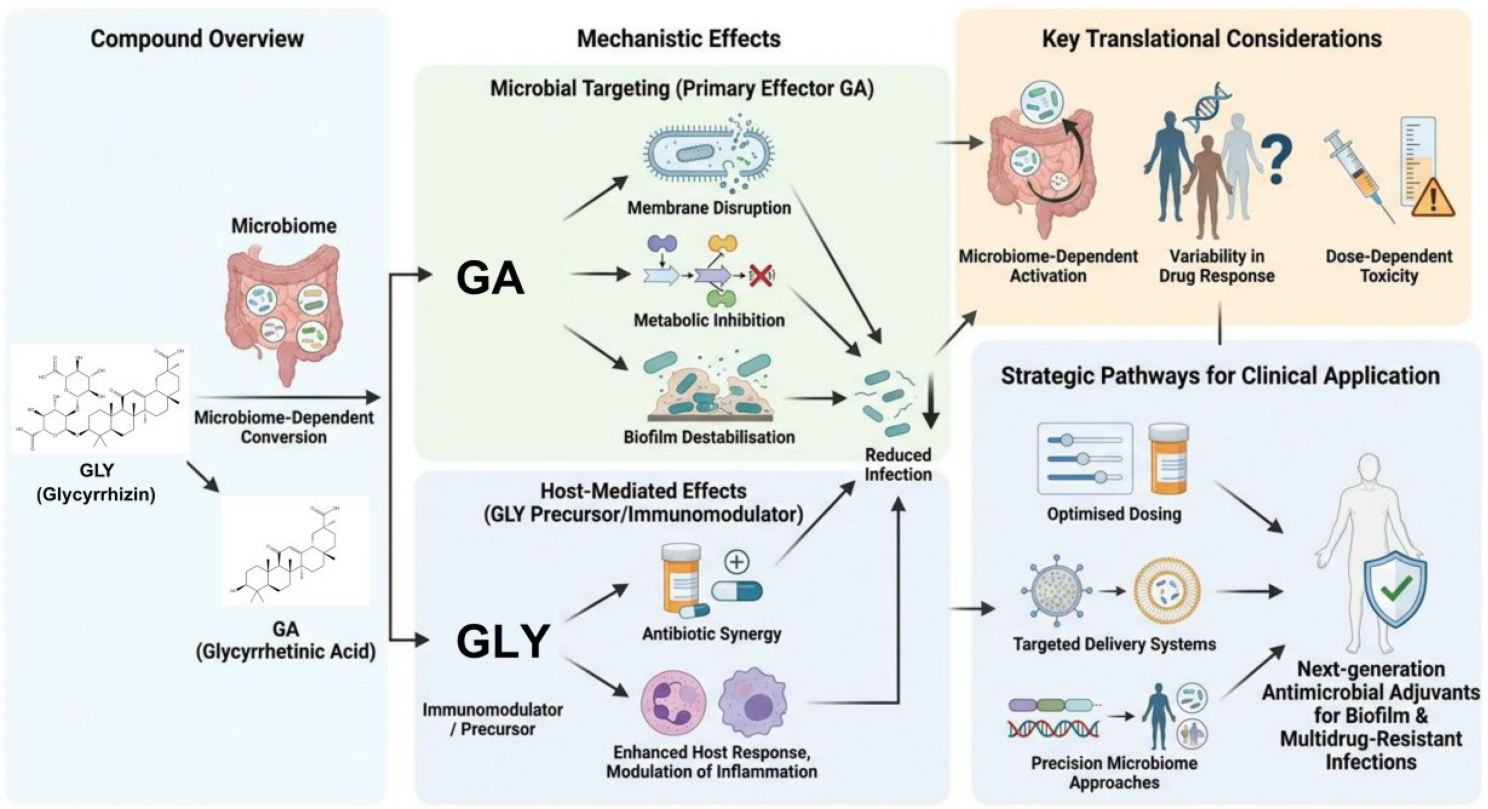
Translational framework and therapeutic outlook of glycyrrhizin (GLY) and glycyrrhetinic acid (GA) triterpenoids. This schematic summarizes the integrated antimicrobial potential of GLY and GA, combining direct microbial targeting with host-mediated effects. GA is depicted as the primary effector driving membrane disruption, metabolic inhibition, and biofilm destabilization, while GLY functions as a precursor and immunomodulator, enhancing antibiotic synergy. The Figure highlights key translational considerations, including microbiome-dependent activation, variability in drug response, and dose-dependent toxicity. Strategic pathways for clinical application—such as optimized dosing, targeted delivery systems, and integration with precision microbiome approaches—are illustrated to demonstrate how these compounds may be developed into next-generation antimicrobial adjuvants for biofilm-associated and multidrug-resistant infections.

The pharmacokinetic profiles of GLY and GA triterpenoids are central to their function as multi-target antimicrobial agents (Table 1). GLY acts as a microbiome-activated prodrug, undergoing bacterial hydrolysis in the gut to yield GA, thereby linking drug activation directly to host microbial ecology (Ishida et al., 2022; Shi et al., 2025). This dependency introduces variability in systemic exposure (Cao et al., 2022; Ploeger et al., 2001) but also highlights a unique opportunity to integrate microbiome-informed strategies into antimicrobial therapy (Figure 5). Once formed, GA is readily absorbed and undergoes enterohepatic recirculation, resulting in sustained systemic levels that are advantageous for targeting persistent and biofilm-associated infections (Chen L. et al., 2024).

In the context of antimicrobial resistance, these pharmacokinetic features support prolonged interaction with microbial communities and infected tissues, reinforcing the multi-target activity described in earlier sections. GA exerts direct effects on bacterial cells, including membrane destabilization, metabolic disruption, and interference with biofilm formation and maintenance. These actions reduce structural integrity and resilience of biofilms, enhancing antibiotic penetration and restoring susceptibility in resistant populations (Dewake et al., 2021; Weaver et al., 2022). Complementing this, GLY contributes host-directed effects by modulating inflammatory signaling pathways such as HMGB1 and NF-κB, thereby promoting pathogen clearance while limiting excessive inflammation (Chen L. et al., 2024; Chen R. et al., 2024; Semwal et al., 2025).

These combined antimicrobial, antibiofilm, and immunomodulatory activities underpin the strong translational potential of GLY and GA as adjuvant therapies in antimicrobial resistance (Figure 5). Despite these promising attributes, translation into clinical practice remains limited by the lack of large-scale clinical validation studies and standardized therapeutic frameworks (Chen L. et al., 2024; Chen R. et al., 2024; Ploeger et al., 2001). Current evidence is largely derived from *in vitro* and preclinical models, and therefore may not fully capture the complexity of host–pathogen interactions in human infections. In addition, variability in bioavailability and microbiome-dependent metabolism poses challenges for achieving consistent therapeutic dosing across patient populations (Ishida et al., 2022). These constraints highlight the need for rigorous clinical trials and pharmacodynamic optimisation. Their ability to weaken biofilm defenses and enhance host responses creates a synergistic framework when used alongside conventional antibiotics, particularly against multidrug-resistant pathogens.

However, this potential is constrained by safety considerations. GA inhibits 11β-hydroxysteroid dehydrogenase type 2 (11β-HSD2), leading to cortisol-mediated activation of mineralocorticoid receptors and subsequent sodium retention, hypokalaemia, and hypertension (Kosicka et al., 2013; Quaschning et al., 2001; Van Uum et al., 2002). These dose-dependent effects necessitate careful optimisation of dosing strategies and limit long-term or high-dose systemic use (Ma et al., 2026). Variability in microbiome-driven activation further complicates predictability of responses across patient populations.

Future translational advances should focus on targeted delivery systems, structural optimisation, and microbiome-informed dosing frameworks to improve therapeutic precision while minimizing toxicity (Mittal et al., 2023). By addressing these limitations, GLY and GA can be more effectively positioned within next-generation multi-target antimicrobials (Figure 5).

## Conclusion and outlook

Both triterpenoids, GLY and GA represent together a multi-target antimicrobial platform that integrates three critical dimensions of infection control: direct antimicrobial activity, biofilm disruption, and host immune modulation (Figure 5). GA primarily targets microbial physiology by disrupting membranes, altering metabolism, and destabilizing biofilm architecture, while GLY complements these effects through microbiome-dependent activation and regulation of host inflammatory responses.

This coordinated functionality provides a systems-level approach uniquely suited to combat antimicrobial resistance, particularly in biofilm-driven and multidrug-resistant infections where conventional antibiotics alone are insufficient. Importantly, their greatest promise lies not as standalone agents but as synergistic adjuvants capable of restoring antibiotic efficacy and enhancing host-mediated clearance.

Despite this potential, successful clinical translation will require overcoming key challenges, including microbiome-driven pharmacokinetic variability, safety limitations associated with GA, and the need for robust validation in clinically relevant infection models. Addressing these issues will require integration of precision microbiome profiling, standardized biofilm models, and advanced drug delivery strategies. Nevertheless, it is important to recognize that the current evidence base remains incomplete, particularly with respect to long-term safety, pharmacodynamic consistency, and efficacy in complex clinical infections (Cao et al., 2022; Ma et al., 2026; Van Uum et al., 2002).

While GLY and GA demonstrate strong antimicrobial and antibiofilm activity in controlled experimental settings, their translation into routine clinical use requires careful balancing of therapeutic benefits against potential adverse effects, including endocrine disruption and interindividual variability in drug metabolism. With these developments, GLY and GA could well be positioned to emerge as next-generation antimicrobial adjuvants, bridging microbial eradication and host-directed therapy and offering a comprehensive strategy to counteract drug-resistant pathogens.
